# How to train your myeloid cells: a way forward for helminth vaccines?

**DOI:** 10.3389/fimmu.2023.1163364

**Published:** 2023-05-30

**Authors:** Rory Doolan, Namitha Putananickal, Lucienne Tritten, Tiffany Bouchery

**Affiliations:** ^1^ Medical Parasitology and Infection Biology, Swiss Tropical and Public Health Institute, Allschwil, Switzerland; ^2^ University of Basel, Basel, Switzerland; ^3^ Institute of Parasitology, Vetsuisse Faculty, University of Zurich, Zurich, Switzerland

**Keywords:** trained immunity, helminth, myeloid cells, vaccine, cellular immunity

## Abstract

Soil-transmitted helminths affect approximately 1.5 billion people worldwide. However, as no vaccine is currently available for humans, the current strategy for elimination as a public health problem relies on preventive chemotherapy. Despite more than 20 years of intense research effort, the development of human helminth vaccines (HHVs) has not yet come to fruition. Current vaccine development focuses on peptide antigens that trigger strong humoral immunity, with the goal of generating neutralizing antibodies against key parasite molecules. Notably, this approach aims to reduce the pathology of infection, not worm burden, with only partial protection observed in laboratory models. In addition to the typical translational hurdles that vaccines struggle to overcome, HHVs face several challenges (1): helminth infections have been associated with poor vaccine responses in endemic countries, probably due to the strong immunomodulation caused by these parasites, and (2) the target population displays pre-existing type 2 immune responses to helminth products, increasing the likelihood of adverse events such as allergy or anaphylaxis. We argue that such traditional vaccines are unlikely to be successful on their own and that, based on laboratory models, mucosal and cellular-based vaccines could be a way to move forward in the fight against helminth infection. Here, we review the evidence for the role of innate immune cells, specifically the myeloid compartment, in controlling helminth infections. We explore how the parasite may reprogram myeloid cells to avoid killing, notably using excretory/secretory (ES) proteins and extracellular vesicles (EVs). Finally, learning from the field of tuberculosis, we will discuss how anti-helminth innate memory could be harnessed in a mucosal-trained immunity-based vaccine.

## 1 Introduction

Helminth parasites are pathogens of importance to humans and the field of veterinary medicine. Despite its large socioeconomic burden, the mass drug administration of anthelmintics is currently the preferred method of control. However, with the rapid emergence of genetic resistance to anthelmintics, such as macrocyclic lactones in livestock, a more sustainable control strategy for helminths is required (1, 2). Vaccination is considered to be the most feasible strategy for the long-term control of such pathogens. However, to date, there is no vaccine for helminth control in human populations, and only a few have been licensed for use against soil-transmitted helminths in agriculture. Irradiated larval vaccines can effectively protect against *Dictyocaulus viviparus* (3), the bovine lungworm, and a native antigen vaccine is available for *Haemonchus contortus* (4), the barber’s pole worm found in sheep and goats, and is in use principally in Australia. Recombinant vaccines have also been developed against *Taenia solium* in pigs and *Echinococcus granulosus* in cattle. The development of these vaccines and the challenges of translating the lessons learned from pastoral to clinical settings have been recently reviewed (5, 6).

However, it is important to note that, despite the efficacy of agricultural helminth vaccines, their recommended vaccination schedule fails to meet the target product profile (TPP) of the human helminth vaccines (HHVs) currently in development. The TPPs for two separate HHVs, hookworm, and schistosomiasis, include characteristics that would make them suitable for use in endemic and resource-limited settings: a maximum of two doses (specifically *via* an intramuscular route for hookworm); an efficacy of 80% for moderate-to-severe hookworm infections and 75% for schistosome burdens; with sustained protection against schistosomiasis for 2 or 3 years (7–9). In striking contrast, the sheep vaccine Barbervax, which is used against *H. contortus* (also known as Wirevax in South Africa), requires three priming doses before the prospective worm season and continuous dosing every 6 weeks during all subsequent worm seasons (10, 11). This intensive schedule is thought to be due to its “hidden” antigen design (an approach shared by the human hookworm vaccine initiative), which uses gut-derived worm products against which no pre-existing allergic immunity exists. The immune system is therefore not sufficiently restimulated by hidden antigens during infection, meaning that regular booster doses are necessary. In general, HHVs have faced considerable challenges, as the type 2 immune response required to control helminth infection can also induce adverse events in the form of allergy and anaphylaxis (12), which has given rise to commercial and ethical concerns. Although humoral immunity will likely be important in any HHV that is developed, the current hidden antigen strategy appears to be inadequate for the recall of immune memory (13).

Cell-mediated immunity is often neglected in the field of helminth vaccines, despite innate cells like eosinophils being “textbook” anti-helminth responders, and many protective cellular mechanisms being described in laboratory helminth models. This is in part because of the historical focus on adaptive immune cells in the field of vaccinology. Currently, with a new understanding of “memory” in the form of trained immunity, innate cell immunity could be harnessed alongside humoral responses to develop effective HHVs. In this review, we argue for the important, though often obscured, role of myeloid cells in the control and killing of helminths and the importance of integrating cell-mediated responses into vaccine design.

## 2 Evidence for myeloid cell killing and control of infection

Increased counts of eosinophils, mast cells, and basophils, both in the blood and at infection sites, are considered a hallmark of helminth infection. However, the protective role of myeloid cells, especially eosinophils, is highly controversial and most likely varies depending on the causative species of infection.

### 2.1 Are myeloid cells involved in helminth control?

In this article, we provide recent evidence and information regarding the controversies associated with myeloid cell involvement in helminth killing and also discuss the mechanisms that are responsible for causing parasite death.

#### 2.1.1 Macrophages

Macrophages are one of the most well-studied effector cells in the context of helminth infection. The type 2 cytokines interleukin 4 (IL-4) and IL-13 polarize macrophages into alternatively activated phenotypes (AAMs; also known as type 2 or M2 macrophages), with the hallmark expression of arginase 1 (Arg-1), resistin like alpha (RELMα), and chitinase-like 3 (Ym1, also Chil3), to name a few. It is well established in laboratory models that AAMs are involved in protection against reinfection for many helminth species, such as filariae (14), soil-transmitted helminths (15–17), *Trichinella* (18, 19), and *Schistosoma* (20). How such macrophages effectively kill helminths is not fully understood, but recent discoveries have shed light on a multitude of factors that potentiate AAM “larvicidal” or anti-helminth killing, such as complement protein C1q, efferocytosis of apoptotic neutrophils, and the activity of surfactant proteins (21–23). However, whether AAMs are self-sufficient or require a persistent type 2 cytokine milieu for the long-term maintenance and killing of secondary infections is currently unknown. Type 2 innate lymphoid cells (ILC2s) have been shown to maintain AAMs in the lungs after infection with the so-called rodent “hookworm” *Nippostrongylus brasiliensis* (15), but whether the “memory” is intrinsic to ILC2s or macrophages in this model is unknown.

In recent years, it has been found that macrophages are plastic; their phenotypes are heavily dictated by their developmental origin and the microenvironment of their niche. For example, it has previously been shown that interstitial macrophages can trap and kill *N. brasiliensis* in the lungs during secondary infection (15). Svedberg and colleagues have demonstrated that alveolar macrophages are poorly polarized into AAMs as glucose availability in the airways is limited (24). However, Chen et al. have recently elaborated on these findings, showing that monocyte-derived alveolar macrophages expand after *N. brasiliensis* infection and polarize more efficiently into AAMs than their tissue-resident counterparts, thus contributing to improved helminth killing (25). In the context of filarial infections, the local proliferation of AAMs was previously associated with infection control (14). However, this might be attributable to the tissue studied rather than a helminth-specific difference, as with the same parasite, *L. sigmodonti*s, it was found that alveolar macrophages are expanded through monocyte recruitment, as in the case of *N. brasiliensis* (26).

All in all, these examples show that, despite their apparent role in helminth killing, not all AAMs are equally effective, and understanding the tissue of protection and the origin of activated cells is crucial to orchestrating protection.

#### 2.1.2 Eosinophils

Despite their “textbook” definition, eosinophils play a controversial role in helminth infection. Research half a century ago established that eosinophils can kill helminths, as observed using *in vitro* assays (27). Yet their role *in vivo* remains rather obscure. Indeed, the depletion of eosinophils using IL-5 ablation observed in Δdbl-GATA-1 mice or PHIL mice in various helminth models has not confirmed the protective role of eosinophils (28–31). In filariae, for which this question has been studied in more detail, it has been suggested that eosinophils are required for parasite development and impact viability only at a later time point (*i.e.*, the patent phase) (32, 33). In addition, it has been proposed that they play a strong role in repair mechanisms (34).

Pulmonary eosinophils have recently been shown to be activated distally by infection with the rodent enteric helminths *Heligmosomoides polygyrus bakeri* (*Hpb*) and *Strongyloides venezuelensis* (35, 36). In the former, eosinophils were expanded distally by CD4+ T cells, whereas in the latter model, type 2 innate lymphoid cells (ILC2s) were found to activate eosinophils. However, it was found that in both cases, eosinophils and IL-5, the potent inducer of their effector functions, were required for heterologous protection against infection with a second rodent parasite, *N. brasiliensis*.

Overall, the role of eosinophils in protection against helminths remains unclear. There could be many technical explanations as to why the *in vitro* and *in vivo* data do not seem to align, such as the tissue origin of the eosinophils used for the studies, or the side effects of constitutive depletion of a population that is extremely important for homeostasis (37). Another explanation could be that helminth parasites have evolved such strong evasion strategies against helminths *in vivo* that depletion of certain cells does not impact parasite survival, as we have observed with the *N. brasiliensis* evasion of neutrophil extracellular traps (see Section 2.2).

#### 2.1.3 Mast cells

Evidence regarding the protective role of mast cells during helminth infection is scarce, but their proteases can reportedly damage the nematode cuticle (38). After infection with the strict enteric murine nematode *Hpb*, the intestinal epithelial barrier is breached by third-stage infective larvae (iL3), which undergo a developmental stage in the submucosa. Mast cells lining the mucosa of the small intestine then detect the adenosine triphosphate (ATP) released from the damaged epithelial cells and release IL-33 to rapidly promote the activation of type 2 innate lymphoid cells (ILC2s). The overabundance of mast cells at this mucosal site is sufficient to render mice resistant to *Hpb* infection, demonstrating the importance of myeloid cells in the early detection of helminth parasites (39).

Mast cells also contribute to the elimination of adult worms. For example, during infection with the murine whipworm *Trichuris muris*, it was found that mast cells enhanced intestinal epithelial cell permeability, which promoted the resulting “weep and sweep” response that expels adult worms. In this model, mast cells continue accumulating in the mucosa and actively secrete proteases for more than 28 days after the expulsion of *T. muris* from the intestine (40, 41). Mast cell-dependent expulsion has also been reported for the rat tapeworm *H. diminuta* using *Kit^W-sh^*-deficient mice that displayed delayed worm expulsion (42). Similarly, mast cells have been shown to be essential for adult expulsion using a novel model of mast cell deficiency (in *Cpa3-Cre* mice) that does not affect basophil number, contrary to *c-kit*-deficient mice (43).

Mast cells’ protective activity seems to be mainly directed against adult parasites living in the intestine. It would be interesting to use the new genetic deficiency models to investigate if mast cells play a role against parasites at other stages or in other tissues.

#### 2.1.4 Basophils

Activated basophils are robust producers of the type 2 cytokines IL-4 and IL-13, which are central to type 2 response initiation. Their role seems to be highly dependent on the parasite model used, which has recently been comprehensively reviewed (44). After *Trichinella spiralis* infection, basophils have been shown to be rapidly recruited systematically by thymic stromal lymphopoietin (TSLP)-dependent mechanisms (45). Using Bas-TRECK mice deficient in basophils, the authors further show that basophils are required for optimal T helper cell type 2 (Th2) activation. Interestingly, in *N. brasiliensis* and *Hpb*, two rodent models of hookworm infection, basophils were not shown to be required for the control of primary infection, but their depletion or absence compromised memory against reinfection with poor Th2 priming or poor macrophage polarization (46, 47). Conversely, in a model of another soil-transmitted helminth, *Strongyloides ratti*, basophils were demonstrated to be crucial to the control of primary infection but redundant in memory responses and the initiation of downstream weep and sweep processes (48). In filarial infection, basophil depletion did not affect outcomes in either primary or secondary infection (49).

To our knowledge, direct helminth killing by basophils has not been reported; however, vaccination against Nb-LSA1a, a secreted product of *N. brasiliensis*, was recently found to confer protection in a basophil-dependent manner (50).

There is limited clinical evidence showing the same in humans. In fact, a retrospective analysis of 22 years of patient medical records did not find an association between helminth infection and basophilia (51), although only circulating basophils were measured. Although morphologically similar, caution should be taken when translating functional discoveries from mouse to human myeloid cells.

#### 2.1.6 Neutrophils

Neutrophils are quickly recruited to the site of tissue damage. Given the large size of helminths, neutrophils are often transiently present at the site of migration. In the hookworm model *N. brasiliensis*, the role of neutrophils in tissue repair has been well established (52, 53). Neutrophils have also been shown to be important for the efficient priming of macrophages in the lungs of hookworm-infected mice (16, 25). Azurophilic and tertiary granule release by neutrophils is important for the elimination of *S. ratti* (54). Direct killing of helminths by neutrophils has more recently been proposed in the context of extracellular trap formation (Section 2.2.3 contains further details).

### 2.2 Mechanisms of helminth killing mediated by myeloid cells

**Figure 1:**
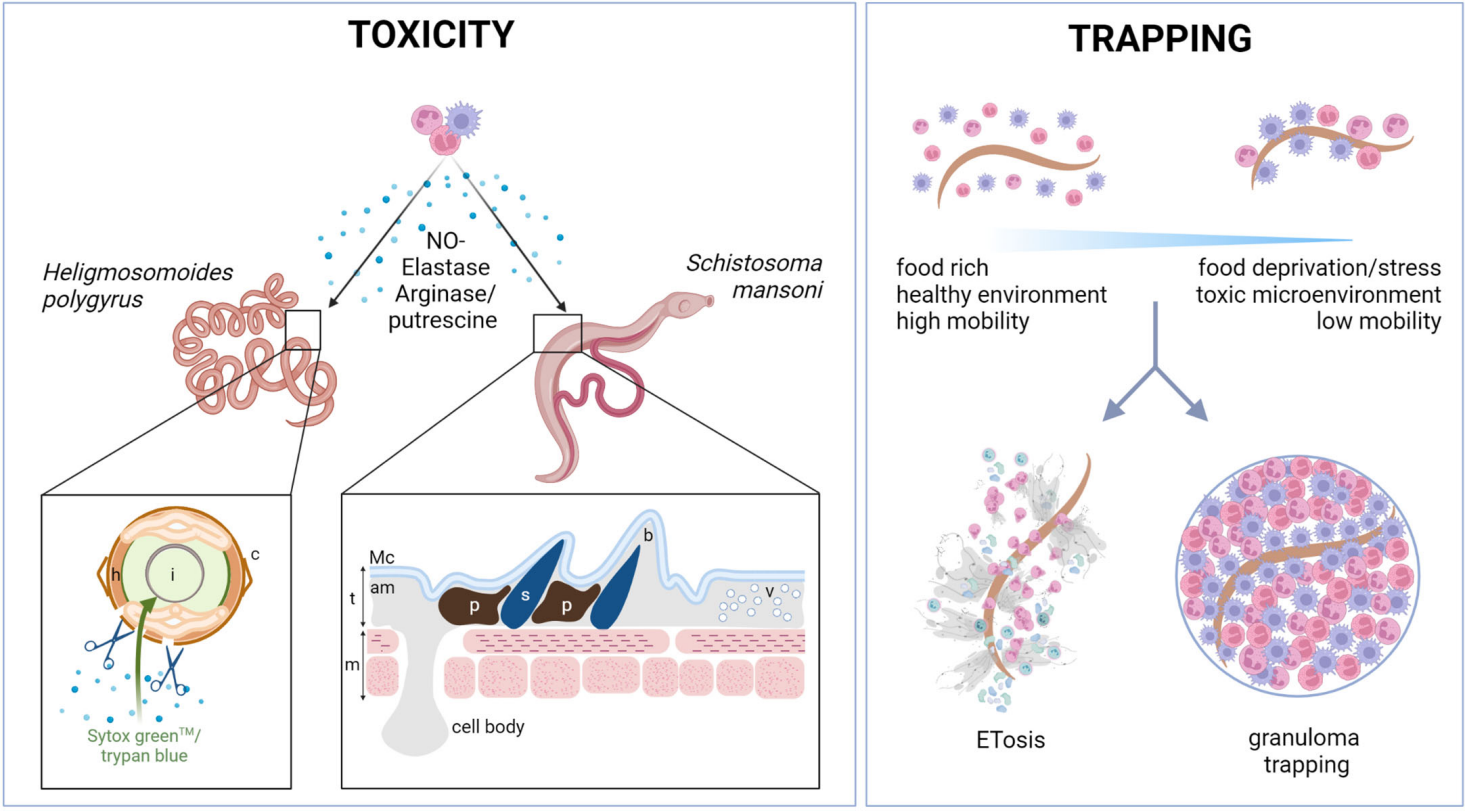
Myeloid cells kill helminths by releasing toxic compounds or by trapping them. Myeloid cells can kill helminths directly by secreting toxic compounds. In nematodes such as *Hpb*, toxic compounds released by myeloid cells, such as putrescine or elastase, can act directly on the parasite cuticle (c), increasing its permeability to viability dyes (such as trypan blue or Sytox Green™) and reducing parasite motility. In trematodes, such as *S. mansoni*, the tegument structure (t) can be affected by neutrophils and eosinophils, as shown by TEM analysis (55). The appearance of dark pigmentation and vesicles under the apical membrane (am) and before the muscle layer (m) was reported after neutrophil and eosinophil binding. The spine area may also be affected, with a clear “bubble“ area reported around some spines (s). l, lumen; h, hypodermis; t, tegument; m, muscle cells; am, apical membrane; Mc, membranocalyx plasma membrane; b, “bubble” area; p, pigment; v, vesicles; TEM, transmission electron microscopy. Another key mechanism of helminth trapping is either by granuloma formation or by extracellular trap formation (ETosis). Trapping provides a way of immobilizing parasites causing food deprivation, stress, and the formation of a potentially toxic microenvironment. Extracellular traps have recently been reported in response to many helminths and can be formed by neutrophils, macrophages, and eosinophils, among other cells. The proteins that decorate the traps are likely to be dependent on the cell types and activation phenotypes of the myeloid cells involved. Physical trapping and close proximity to toxic molecules have been shown to result in larval death. Trapping can also take the form of granulomas, the composition of which varies between helminth species. Typically, type 2 granulomas are rich in macrophages and eosinophils, and occasionally, in neutrophils. Granulomas are usually surrounded by structural cells such as epithelial cells and fibroblasts. Their role remains poorly understood. Figure created using BioRender.com.

Three main mechanisms of helminth killing orchestrated by myeloid cells have been described: (i) direct toxicity of released mediators, (ii) killing by trapping in granulomas, and (iii) killing by extracellular trap formation (**Figure 1**).

#### 2.2.1 Toxicity

Many studies report the involvement of myeloid cell-derived enzymes and cationic/cytotoxic proteins in helminth infection.

For example, Arg-1, released by macrophages, hydrolyzes the amino acid arginine to ornithine and putrescine, which can directly immobilize *Hpb* (17) (**Figure 1**). Eosinophil granule proteins are also produced and secreted, including eosinophil peroxidase (EPX), major basic protein (MBP), eosinophil cationic protein (ECP), and eosinophil-derived neurotoxin (EDN) (56, 57). Notably, several older studies have shown the direct *in vitro* toxicity of these compounds against helminths, such as all four main eosinophil granule proteins against the microfilariae of *Brugia malayi* and *Brugia pahangi* (58). Furthermore, in the 1980s, Jong and colleagues observed that human neutrophils could attach to and kill *S. mansoni* schistosomula when exogenous eosinophil peroxidase (EPX) was added to *in vitro* cultures (59). However, there is little recent literature on the cytotoxic mechanisms of helminth killing.

Other products may not be directly toxic but instead cause damage leading to secondary effects. For example, histamine released from mast cells has long been thought to cause an itch that results in the physical removal of skin-penetrating parasites (60). In the last few years, nociception has been shown to communicate immunological stimuli from distal sites to central lymphoid organs. Cytokines and mediators can innervate peripheral nerves, which go on to influence dendritic cells (DCs) function and germinal center reactions, and this function can be inhibited by non-steroidal anti-inflammatory drugs (61). Indeed, nociceptive neurons may drive immune responses to fungal infections, such as *Candida albicans*, by secreting the neuropeptide calcitonin gene-related peptide (CGRP), which facilitates the production of IL-23 by dendritic cells; in turn, IL-23 activates Th17 cells (62, 63). However, similar mechanisms linked to itching, or pruritus, caused by helminths or other parasites have, to our knowledge, not yet been explored. Instead, much research has focused on the immobilization of infective and adult stages of helminths.

#### 2.2.2 Trapping of helminths: Direct adhesion and granuloma formation

The adhesion of immune cells to the parasite surface has long been observed, and, strikingly, immune cells can even recognize non-parasitic helminths, as demonstrated by eosinophils binding to *Caenorhabditis elegans* L3 (64). Early immunization studies observed that *N. brasiliensis* larvae injected into the peritoneal cavity of naive rats were rapidly coated by macrophages, and although the larvae were not directly damaged by the cells, immobilization appeared to trigger autolysis of the larvae, followed by the death of the rats (65). Another similar study found that the initial response to intraperitoneal injection of this gastrointestinal nematode triggered neutrophilia for several hours, followed by several days of eosinophilia in the peritoneal exudate (66). Since these studies, almost five decades ago, research into granulomatous responses to infection has increased. In general, granulomas are rich in macrophages and eosinophils, with possible recruitment of neutrophils depending on the parasite stage. Granulomas are often surrounded by fibroblasts and/or epithelial cells. Intriguingly, however, there have been few advances in the research on how tissue-dwelling parasites actually meet their end. Immobilization leading to stress and starvation is a common hypothesis for how parasites are destroyed (**Figure 1**).

Recently, arginine depletion in the granuloma microenvironment was explored as a potential explanation for decreased viability due to AAMs trapping, as helminths cannot produce their arginine. *In vitro*, the supernatant of AAMs cocultured with the larvae of *N. brasiliensis* is reduced in arginine. The authors also showed that arginine supplementation can limit the reduction in viability (as measured by ATP concentrations) seen in the parasite after co-incubation with AAMs and that larvae cultured in the absence of arginine (without macrophages) were less viable. Although this amino acid starvation mechanism has not been proven *in vivo*, it certainly consolidates the food deprivation hypothesis (25).

More recently, another form of parasite trapping has been revealed as a mechanism resulting in helminth death.

#### 2.2.3 Trapping of helminths: Extracellular traps

The previous belief that neutrophils are not involved in anti-helminth immunity has fallen out of favor following findings that many helminth species have instead simply evolved mechanisms to evade neutrophil functions altogether.

A key mechanism involves the formation of neutrophil extracellular traps (NETs), so named for being first observed to form in neutrophils. This process involves the release of DNA in the form of decondensed filaments in response to large multicellular pathogens, including fungal hyphae and helminths. These structures are decorated with toxic compounds, including histones and the granule contents of granulocytes, and are derived from either genomic or mitochondrial DNA, giving rise to “suicidal” or “vital” traps, respectively. NETs, or as we refer to them more accurately here, extracellular traps (ETs), can also be produced by eosinophils, basophils, and macrophages, among other cells (67). Thus, many cells can form traps, but for helminth infection in mammals, neutrophil and eosinophil traps (NETs and EETs, respectively) have been mainly described (**Figure 1**). ET formation has been shown to be an ancient form of pathogen defense that predates the development of the mammalian myeloid lineage (68). This is likely a key reason for helminths developing a plethora of evasion strategies against ET formation (ETosis). Such mechanisms will be discussed in a later section. The role of ETosis in helminth infection has recently been reviewed by us (69), and also by Ajendra (70), and, as such, we will mostly focus on the more recent literature.

In a seminal paper, Bonne-Année established that *Strongyloides stercolaris* larvae could trigger the formation of ETs from neutrophils and macrophages *in vitro* (71). However, no killing was observed in this model. Such trapping without killing has been demonstrated for numerous helminth species, both of human and agricultural importance, such as *H. contortus* (72, 73), *B. malayi* (74), *Dirofilaria immitis* (75), *Onchocerca volvulus* (76), *Ostertagia ostertagi* (77), *Fasciola hepatica* (78), *Oesophagostomum columbianum* (79), and *E. granulosus* (80). Interestingly, it was reported that human neutrophils do not release NETs when stimulated by *S. mansoni* eggs but do when stimulated by *S. japonicum* eggs (81). In 2020, we demonstrated, both *in vivo* and *in vitro*, that hookworms can evade NETs by releasing type II deoxyribonuclease II (DNase II), and, that when this evasion strategy was compromised, NETs can impair parasite viability (82). Since then, many mechanisms of ET evasion by helminths have been described (Section 2.2). Thus, earlier publications on the subject may have overlooked ETosis due to such evasion strategies.

Another form of ETosis has been described more recently in the context of helminth infection. Eosinophil ETs against helminths were recently found to form around the microfilariae (mf) of the filarial model *L. sigmodontis*, although not at the L3 stage (83). Mitochondrial markers were found to be higher than nuclear markers in the released structure, indicative of “vital” EETs. Both cavity thoracic and intestinal eosinophils were shown to decrease parasite motility by EETs, and the inoculation of mf pretreated with eosinophils accelerated their clearance *in vivo*. A specific killing mechanism was not further investigated, but eosinophil traps contained ECP, a highly basic cytotoxic RNase found in the granules of eosinophils that have previously been established as toxic for *Brugia* mfs (58).

The actual killing mechanism of helminths by ETs has not been described in detail so far. Trapping has certainly been reported for many species and, as described for granulomas, can impair development and motility. Different shapes of NETs have been reported for the canine filariae *D. immitis*, for example, diffused NETs, spread NETs, and aggregated NETs, all of which were observed after contact with *D. immitis.* It is unclear whether or not those different shapes could have different impacts on worms; however, all of them did promote larval entrapment (75). NETs may not be able to create food deprivation for large helminths; however, they can certainly create a toxic microenvironment that localizes toxic molecules to the parasite, as previously reported in the case of EETs and ECP against mfs.

In general, the molecular triggers of ETosis continue to be defined, but some recognition receptors have been identified. The release of EETs in response to *L. sigmodontis* mfs was shown to be dependent on the recognition receptor dectin-1 but not on either dectin-2 or Mincle (macrophage-inducible C-type lectin) (83). Furthermore, trap formation around *Schistosoma japonicum* was reported to be driven by host-derived extracellular vesicles (EVs) (84), possibly as a host-derived response to overcome immunomodulation by *S. japonicum* through IL-10 regulation. In addition, Ehrens and colleagues have demonstrated that antibodies from immune animals can enhance ETosis, but are not essential for trapping the infectious larvae of soil-transmitted helminths (54).

## 3 Immunomodulation of myeloid cells

During the last decades, research on helminths has shed light on their elaborate strategies for immunomodulation and evasion, which are required for long-lived associations with their hosts (85). Helminths have been proposed to be a driving force in the evolution of type 2 immunity, which balances parasite control with wound healing (86). Because of this, research has broadly studied numerous parasite products involved in host–helminth interactions, particularly those that polarize DCs (and the ensuing CD4^+^ T helper cell responses) and macrophages. Other myeloid cells also appear to be targeted by ES.

**Figure 2:**
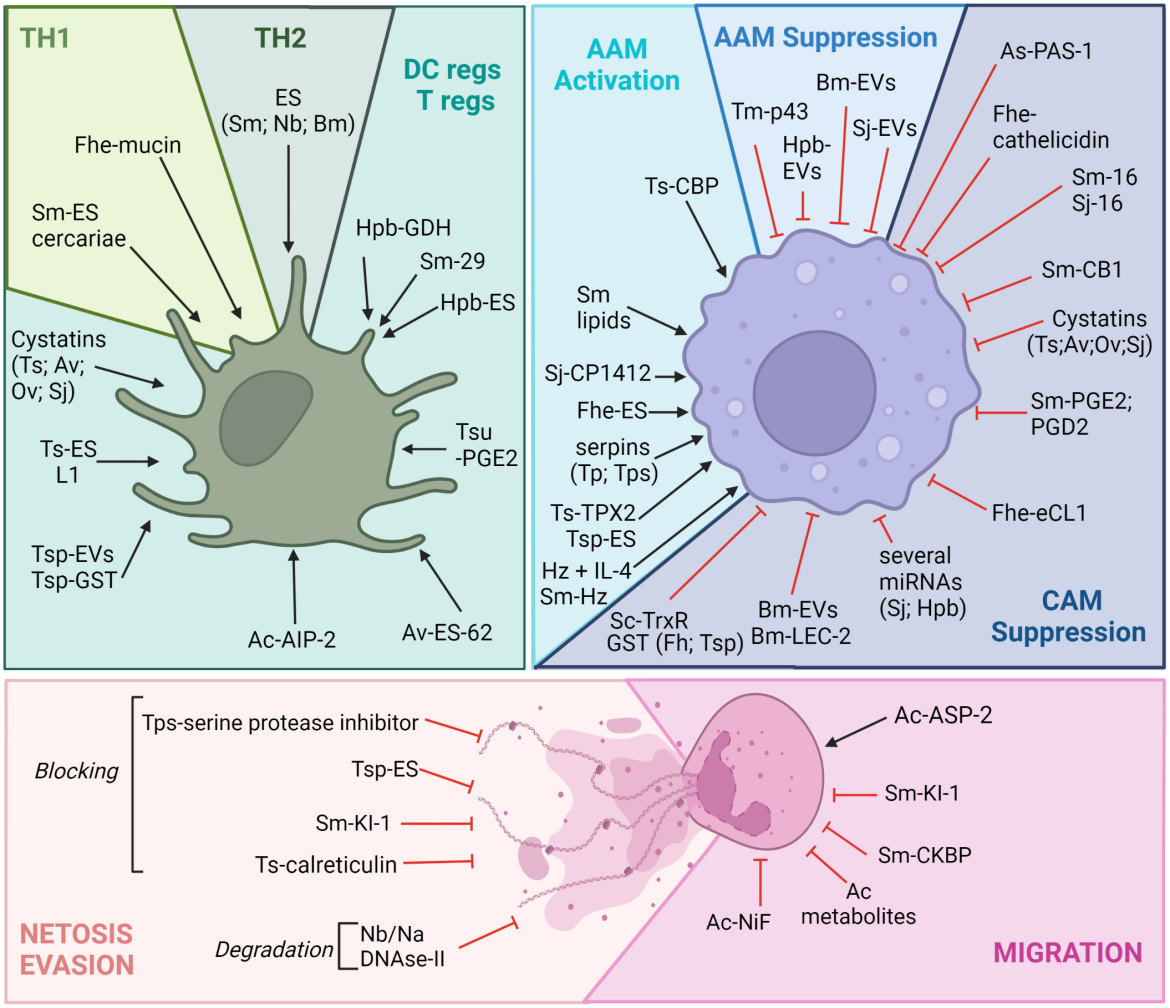
Helminths can immunomodulate myeloid cells to avoid killing. Myeloid cells are particular targets of helminth parasite evasion. Of note is that many ES products have been shown to trigger regulatory phenotypes in DCs and in macrophages, allowing the long-term survival of helminths in their hosts. Many of these immunomodulatory compounds are secreted and can be proteins, metabolites, nucleic acids, EVs, etc. It should be noted that the effects of these products are always ambiguous, as they can trigger both tolerance and regulatory mechanisms, in addition to pro-killing mechanisms (Th2 polarization induced by Sm or Nb products for example through DC activation or the hookworm factor ASP-2, which favors neutrophil migration). Given a large number of evasion molecules, only a few will be presented. Understanding this complex immunomodulation is required for efficient vaccine design, and this figure highlights how myeloid cells are central to this strategy. Ac, *Ancylostoma caninum*; Av, *Acanthocheilonema viteae*; Bm, *Brugia malayi*; Fhe, *Fasciola hepatica*; Hpb, *Heligmosomoides polygyrus bakeri*; Na, *Necator americanus;* Nb, *Nippostrongylus brasiliensis*; Sj, *Schistosoma japonicum*; Sm, *Schistosoma mansoni*, *Of, Opisthorchis felineus*, Ov, *Opisthorchis viverrini*; Tm, *Trichuris muris*; Tp, *Trichinella pseudospiralis*; Tsp, *Trichinella spiralis*, Tsu, *Trichuris suis.* Ag, antigen; AIP, anti-inflammatory protein; ASP-2, aspartic protease 2; CB1, cannabinoid receptor type 1; CkBP, chemokine-binding protein; CBP, cathepsin B-like protein; NiF, neutrophil inhibitory factor; eCL1, extender of the chronological lifespan protein 1; ES, excretory/secretory products; EVs, extracellular vesicles; GDH, glutamate dehydrogenase; GST, glutathione *S*-transferase; IL, interleukin; LEC-2, lectin 2; PGD2, prostaglandin D_2_; PGE2, prostaglandin E_2_; KI-1, Kunitz-type serine protease inhibitor; TPX2, thioredoxin peroxidase 2; TrxR, thioredoxin reductase. Figure created using BioRender.com.

Immunomodulation by ES products of helminths has already been extensively reviewed (85), and, as such, the list provided here is not exhaustive but targeted to highlight the importance of understanding such evasion mechanisms for vaccine design for different myeloid cells, either involved in type 2 polarization or directly as effector cells. ES products are composed of a large array of molecules, such as proteins but also lipids, glycans, other metabolites, and larger structures that contain nucleic acids, such as EVs. In this section, we will discuss some of the key helminth products that mediate the immunomodulation of myeloid cells. It should be noted that only the most recent findings are detailed (**Figure 2**), notably those related to metabolites and EV modulation; descriptions of other mechanisms are available in **Table 1**.

**Table 1:** Helminths can immunomodulate myeloid cells to avoid killing.

Product	Cell target	Main finding	Reference
Hpb-ES	Intestinal DCs	Decreases in surface expression of dectin-1 *in vitro* and *in vivo*	(87)
Nb/Sm-ES	DCs	Th2	(88–90)
Tsp-/Fhe-ES	Macrophages	AAM activation with increased arginase-1 expression	(19, 91)
Tsp-ES	DCs	Impairs maturation and function: low expression of HLA-DR (Human Leukocyte Antigen – DR isotype) , CD86, and CD83	(92)
1—Proteases and inhibitors			
CPIs/Tsp-CstN	BMDM	Downregulates classic activation markers after LPS stimulation *in vitro* by inhibiting the TLR2/MyD88 signaling pathway	(93–98)
Fhe-CL1 and Sm-CB1	Macrophages	Protects in an LPS model by suppressing pro-inflammatory macrophage responses through TLR-3 degradation	(99)
Hpb-CPI	DCs	Tolerogenic activation when stimulated *in vitro* with Hpb-CPI and CpG	(100)
Sj-CP1412	RAW264.7 cells	RNAse, induces AAM polarization	(101)
	DCs	Inhibits LPS-stimulated DC maturation	
Tsp-CBP	Macrophages	Induces AAM polarization to ameliorate intestinal injury in an intestinal ischemia-reperfusion model	(100 ,102)
Tsp-TPX2 released by muscle larvae	RAW264.7 cells/peritoneal macrophages	Upregulates arginase-1 and MRC-1	(103)
Tp-serpins	Macrophages	Inhibits the activity of different proteases, such as human neutrophil elastase, mouse monocyte chemoattractant protein 1, and human neutrophil cathepsin G	(104)
Tp-serpins	J774A.1 macrophages	*In vitro* upregulation of gene expression for the AAM markers IL-10 and *arginase-1*	(104)
Tsp-serpins	intestinal Macrophages	Reduces colitis inflammation by increasing AAM polarization *in vivo*	(105)
2—Antioxidants			
Fhe-GST (omega class)	Macrophage	Increases apoptosis and limits the production of pro-inflammatory cytokines	(106)
Sc-TrxR	Macrophages	Limits the pro-inflammatory cytokine response after LPS stimulation	(107, 108)
Tsp-GST	DCs	Decreases LPS response and increases production of regulatory cytokines IL-10 and TGF-β	(109)
3—Other proteins			
Ac-AIP-2	DCs	Reduces allergic asthma by expanding tolerogenic CD103^+^ DCs	(110)
Bm-LEC2	THP-1 cells	Major EV protein, homologous to mammalian galectin 9, Induces AAM activation	(111, 112)
Hpb-GDH	BMDM, Granulocytes and human PBMCs	Alters the *in vitro* eicosanoid production	(113)
Sm-29	DCs	Reduces both type 1 and type 2 inflammatory responses due to tolerogenic DC expansion in a large variety of experimental models	Reviewed in (114)
Tm-p43	Peritoneal and pulmonary macrophages	IL-13-binding protein, limits AAM differentiation	(115)
Tm-p43	DCs	Increases in MHCII and CD86 expression following pulsing with p43	(115)
Tso-GDH/-IDH	Monocytes	Polarizes peripheral blood monocytes toward tolerogenicity, through regulation of host PGE2	(116)
Tsp-MIF	Monocytes	Binds to and interacts with host monocytes *via* the surface molecule CD74, causing cell proliferation	(117)
4—Metabolites			
Ac-metabolites	PBMCs	Suppress the secretion of inflammatory cytokine	(118)
		Protect in a mouse model of colitis	
4–1 Lipids			
Av-Es-62	Macrophages and DCs	Blocks TLR-4 signaling through its PC moieties	(119, 120)
Sm-LPC	BMDM	Increases expression of Arg-1 mannose receptor, Ym1, TGF-β IL.10, and PGE2 Alternative activation of macrophages through PPAR-γ activation	(121)
Sm-PGE2/PGD2	Macrophages and DCs	Alters macrophage and DC polarization	(122)
Tsu-ES	Human DCs	Decreases secretion of TNF-α after *in vitro* LPS activation, Release of eicosanoids may be involved in further host modulation and suppression of DC response	(123)
4–2 Hemozoin (Hz)			
Of-Hz	Human DCs from bronchial asthma patients	Causes release of IL-10 and IL-12 instead of TNF-α and TGF-β, but no change in DC activation markers	(124)
Sm-Hz	Macrophages/BMDMs	Potentiates IL-4 stimulation and induces alternatively activated macrophage polarization	(125)
5—Extracellular vesicles/helminth-derived extracellular vesicles (EVs)			
Bm-EVs	Macrophages	Causes release of Bm-MIF and promotes AAM by synergizing with IL-4	(111, 112)
Hpb-TGM	Fox3^+^ Treg cells	Induces Foxp3^+^ Treg cells to interact with TGF-β receptor	(126)
Tsp-EVs	Macrophages	Polarizes to AAM M2b subtype	(127)
Tsp-EVs		Prevents colitis by inhibiting CAM polarization and instead increases gut infiltration of AAM cells	(128)
5–1 Evasion from extracellular traps			
Nb/Na-DNase II	Neutrophils	Degradation of NETs	(82)
Sm-KI-1	Neutrophils	Binds to neutrophil elastase and impairs neutrophil migration and function	(129–131)
Tsp-CRT		Reduces ETs formation triggered by albumin/anti–albumin complexes *in vitro*	(132)
Tsp-EVs	Neutrophils	Blocks NETosis	(133)
Tsp-serpin	Neutrophils	Blocks neutrophil elastase and impairs phagocytosis and NETosis Reduces release of pro-inflammatory cytokines and chemokines	(97)
5–2 Evasion from cell recruitment and effector molecules			
Ac-NiF	Neutrophils	Inhibits neutrophil accumulation at sites of tissue injury through CD11b/CD18 binding	(134)
Ac-ASP-2	Neutrophils/monocytes	Recruits neutrophils and monocytes (skin)	(135)
PAS-1	Eosinophils	anti-inflammatory properties Impairs EPX activity and reduces levels of IL-4, IL-5, IL-13 and eotaxin	(136–138)
Av-ES-62	mast cells/DC regs/Tregs	Inhibits mast cell degranulation *via* its ability to “reset” homeostatic signaling of ST2	(139)
Sm-LPC	Human eosinophils	Activates lipid droplet biogenesis *via* TLR2 *in vitro*	(140, 141)

AAM, alternatively activated phenotype; BMDM, bone marrow-derived macrophage; CAM, classical activation of macrophage; DCs, dendritic cells; ETosis, ET formation; ETs, extracellular traps; EVs, extracellular vesicles; EPX, peroxidase; IL, interleukin; miRNAs, microRNAs; LPS, lipopolysaccharides; NETs, neutrophil extracellular traps; NETosis, NET formation; PBMCs, peripheral blood mononuclear cells; PPAR-γ, peroxisome proliferator-activated receptor gamma; TGF-β, transforming growth factor beta; Tregs, T-regulatory cells; TNF-α, tumor necrosis factor alpha; PC, phosphorylcholine.

Helminths have evolved many evasion strategies directed against myeloid cells, either by polarization or effector mechanisms. Some examples are listed in the table, which represents all major classes of secreted products. Ac, *Ancylostoma caninum*, Av, *Acanthocheilonema viteae*; Bm, *Brugia malayi*; Fhe, *Fasciola hepatica*; Hpb, *Heligmosmoides polygyrus bakeri*; Na, *Necator americanus*; Nb, *Nippostrongylus brasiliensis*; Of, *Opisthorchis felineus*; Sj, *Schistosoma japonicum*; Sm, *Schistosoma mansoni*; Sc, *Setaria cervi*; Tm, *Trichuris muris*; Tp, *Trichinella pseudospiralis*; Tso, *Taenia solium*; Tsp, *Trichinella spiralis*; Tsu, *Trichuris suis*; AIP-2, anti-inflammatory protein 2; BMDM, bone marrow-derived macrophages; cystatins or CPI, cysteine protease inhibitors; FheCL1, a major cysteine protease of Fasciola hepatica; GDH, glutamate dehydrogenase; GST, glutathione S-transferase; IDH, isocitrate dehydrogenase; MHC II, major histocompatibility complex II; MIF, macrophage migration inhibitory factor; PGE2/PGD2, prostaglandin D_2_ or E_2_; Serpins, serine protease inhibitors; SmCB1, major cysteine proteases of *S. mansoni*; Ts-CBP, *T. spiralis* cathepsin B-like protein; TsCstN, *T. spiralis* cystatin; TsTPX2, Tsp-anti-apoptotic protein thioredoxin peroxidase 2; TrxR, thioredoxin reductase; HLA-DR, Human Leukocyte Antigen – DR isotype.

### 3.1 Shaping polarization: Type 2, regulatory or type 1 responses of macrophages and DCs

#### 3.1.1 Helminth secreted proteins

Helminth products exert a strong influence on the immune response and shape type 2, regulatory, and type 1 response. These changes are well studied for both DCs and macrophages, which set the immunological tone of response (142), both triggering a Th2 response (88–90) and shifting the response away from type 1 and promoting the expansion of regulatory T cells (Tregs) (87, 92). Similar to DCs, secreted products from several helminths, notably proteases and their inhibitors, can stimulate macrophages to upregulate hallmark markers of AAMs and alleviate inflammatory diseases (19, 91, 99, 101–105). For example, a range of helminths have evolved to express cystatins, also called cysteine protease inhibitors (CPIs), including *T. spiralis* (TspCstN) (93)*, Hpb* (HpbCPI) (100), *Acanthocheilonema viteae (*AvCystatin) (143), *O. volvulus (*onchocystatin) (94), *B. malayi* (Bm-CPI-2) (95), and *S. japonicum (*SjCystatin) (96). They present very similar activities that impact both macrophage and DC polarization (85, 93, 97, 98, 100).

Antioxidants, such as glutathione *S*-transferases (GSTs), are detoxification enzymes that play an important role in protection against the free radicals generated by host immune cells, decreasing pro-inflammatory responses both in DCs and macrophages (106–109, 144).

Many other proteins secreted by helminths have been shown to shape macrophage or DCs polarization, thus illustrating how ES might inform “natural” or vaccine-induced immunity by modulating antigen-presenting cells (110, 114, 115, 117). Recently, several metabolic enzymes secreted by helminths have been shown to modify host metabolism by altering eicosanoid and prostaglandin production (113, 116).

In summary, the proteins secreted by helminths have both demonstrable and putative influences on a variety of immune cells, including those in the myeloid compartment that orchestrate vaccine-induced immunity.

#### 3.1.2 Secreted helminth metabolites

To date, most studies on the immunomodulatory products of helminths have focused on proteins. However, recent publications have highlighted that secreted metabolites play an important role in host–parasite interactions. Many have been explored for their therapeutic potential as anti-inflammatory drugs.

For example, *Ancylostoma caninum* metabolites have been shown to be protective in a mouse model of colitis. Notably, further analysis has shown that low-molecular-weight metabolites (of ES and somatic origin) suppress inflammatory cytokine secretion from human peripheral blood mononuclear cells, including lipopolysaccharide (LPS)-stimulated myeloid cells (118). Although the responsible metabolites have not yet been identified, the metabolic characterization of various helminth products is now well under way (145–147).

##### 3.1.2.1 Lipids

Helminth lipidomics is still a nascent field (148) and many of the lipid molecules identified by metabolomics are helminth-specific (146). Recently, it has been identified that eicosanoids produced and released by helminths can have immunomodulatory functions. The prostaglandins E_2_ (PGE2) and D_2_ (PGD2) have been identified as secreted products from schistosomes as demonstrating various non-myeloid immunomodulation activities (122), and both have been postulated to alter macrophage and DC polarization. *Trichuris suis* also exerts immunomodulatory effects *via* helminth-derived eicosanoids. Laan et al. identified an ES compound that reduces the secretion of tumor necrosis factor-alpha (TNF-α) in human DCs after *in vitro* LPS activation. Using fractionation, the active lipid was found to be similar in structure to mammalian PGE2. Further metabolomics of Tsu-ES products confirmed the release of a wide range of eicosanoids that could be involved in further host modulation and the suppression of DC responses (123).

Lysophosphatidylcholine (LPC) from *S. mansoni* has recently been shown to activate bone marrow-derived macrophages to increase expression of the Arg-1, mannose receptor, Ym1, and transforming growth factor beta (TGF-β) as well as production of IL-10 and PGE2 24 h after stimulation. The authors further show that Sm-LPC induces alternative activation of macrophages through peroxisome proliferator-activated receptor gamma (PPAR-γ) activation (121). Lipids can also decorate proteins, as is the case with ES-62, a filarial nematode phosphorylcholine (PC)-containing product that has been extensively characterized and shown to be protective in a wide range of inflammatory diseases (119). Mechanistically, it blocks Toll-like receptor 4 (TLR-4) signaling in DCs and macrophages by way of its PC moieties (120).

Overall, the role of lipids in helminth modulation is still poorly understood, but lipids might offer interesting targets once further characterization are available.

##### 3.1.2.2 Hemozoin

Hematophagous helminths, such as schistosomes, liver flukes, and hookworms, can detoxify free heme that is released during hemoglobin digestion by the formation of a polymer of heme called hemozoin (Hz) (149–152). Hz potentiates IL-4 stimulation and induces AAM polarization (125). Interestingly, human DCs isolated from patients with bronchial asthma and then pulsed with LPS and Hz released IL-10 and IL-12 instead of TNF-α and TGF-β, and no change in DC activation markers was observed. Surprisingly, no effect on the human DCs of healthy individuals was noted (124). It could thus be crucial to investigate the role of Hz in DC polarization in the presence of IL-4 or the impact of Hz presence on vaccination.

#### 3.1.3 Helminth EVs and their cargo

EVs are small membrane-bound packages, including both exosomes and microvesicles (153), that are shed by virtually all living cells into their environment, and which can be taken up by and subsequently affect the function of distant cells. The role of EVs in both innate and adaptive immune responses was reviewed recently (154). EV trafficking has emerged as a central mechanism of intercellular communication in mammals and appears to play an important role in host–pathogen interactions (155). Interactions occur either through the activation of receptors located on the surface of recipient cells, and/or through the transfer of membrane-encapsulated cargo. EVs are enriched in molecules that either reflect their subcellular origin or are selectively packaged within them. In most cases, the precise mechanisms by which EVs exert their functions remain to be elucidated. EVs are internalized by many cell types and by multiple pathways (156). Antigen-presenting cells may capture EVs by phagocytosis, followed by endocytosis. In other cells, EVs may be internalized into endolysosomal compartments, where a fraction may escape to release their contents into the cell cytosol by back-fusion with the endosomal membrane (157). Under specific conditions, EVs can fuse directly with the acceptor cell membrane (157, 158).

EVs contain a wide array of molecules, from lipids delimiting their structure to transmembrane and intraluminal proteins and glycoproteins, small metabolites, and nucleic acids (156, 159, 160). Interestingly, in two different infection settings, macrophages were the host cell type where most helminth-derived microRNAs (miRNAs) could be detected *in vivo* (161, 162).

Growing evidence shows that the uptake of helminth-derived EVs leads to the downregulation of immune responses (163). EVs carry proteins with known immunomodulatory properties. A TGF-β mimic (Hpb-TGM) was detected in the EVs of *Hpb* and can induce Foxp3^+^ Treg cells upon interaction with the TGF-β receptor (126). To date, the role of Hpb–TGM in DC and macrophage polarization has not been investigated, but it may impact them in a similar manner to mammalian TGF-β (164). *Brugia malayi* EVs carry other homologs such as macrophage migration inhibitory factor (MIF) (111) and galectin-2, a mammalian galectin-9 homolog (Bm-LEC-2) that promotes the alternative activation of macrophages by synergizing with IL-4 (112). In a mouse model, it was found that *Trichinella spiralis* EVs promote the polarization of macrophages to AAM of the so-called M2b subtype (127). EVs from the same parasite prevent colitis by inhibiting classical (M1) macrophage polarization and increasing the gut infiltration of AAM cells (128). Because helminth EVs have the potential to exert important immunomodulatory effects on host cells, they are considered a rich source of antigens for new vaccines, particularly those based on neutralizing antibodies (163, 165) (Section 4.3.2).

Dozens of helminth ES miRNAs can be found in parasite-infected host blood or culture and are often packaged in EVs (160). MiRNAs are short non-coding RNA molecules that regulate the post-transcriptional expression of a large number of genes, typically resulting in messenger RNA (mRNA) degradation, and the suppression of translation (166). In the past decade, the ability of parasite-derived miRNAs to modulate both innate and adaptive immune responses by being transferred to host cells has become increasingly compelling (167). Both *in vitro* and *in vivo*, pivotal host immune functions are repressed by nematode miRNAs following exposure to miRNA mimics or internalization of parasite-derived EVs (161, 167–176). The evidence so far implies widespread incorporation of helminth miRNAs into a broad range of host cells, including innate cells such as macrophages.

EVs appear to play an important role in host-parasite communication and myeloid cells in particular, as they seem to be a major conduit for such communication.

As we have seen, helminths have evolved a plethora of mechanisms to affect the polarization of DCs and macrophages, pushing the immune system away from protective responses. However, helminth products can also directly block protective cellular mechanisms in various myeloid cells, such as basophils, mast cells, neutrophils, and eosinophils.

### 3.2 Direct evasion from myeloid killing

#### 3.2.1 Evasion from extracellular traps

Research is currently pointing toward an “arms race” of neutrophil-mediated killing and immune evasion. Trap evasion can involve either inhibiting the process of DNA decondensation and release, *i.e.*, inhibiting ETosis, or simply the degradation of released DNA by DNase enzymes. Both mechanisms have now been observed across a wide range of helminths in the last 5 years alone.

Using a live imaging approach, *N. brasiliensis* was shown *in vivo* to have the ability to degrade NETs by the secretion of DNase II (82). A similar evasion mechanism has been confirmed in the human hookworm *Necator americanus in vitro*.

Cyst fluid from *E. granulosus* was found to decrease NET formation against the parasite. At this stage, further mechanisms have not been defined (80). Similarly, *F. hepatica* induced only “weak” NET formation (NETosis) against both juveniles and metacercariae *in vitro* using bovine neutrophils (78). The authors found low reactive oxygen species (ROS) production, but this mechanism was not investigated further. ES from *Mesocestoides corti* (a model of *Taenia* spp.) was found to inhibit hydrogen peroxide (H_2_O_2_)-induced NET formation both *in vitro* and *in vivo* in a sepsis model of NETosis. The authors have further shown that Mc-ES inhibits ROS-induced NET formation by blocking the non-selective calcium-permeable channel TRPM2 (transient receptor potential cation channel subfamily M member 2) channel and calcium ion (Ca^2+^) entry (177).

Recently, *T. spiralis* ES products were shown to block NETosis. The decondensation of DNA and citrullination of histones took place regardless of the stimuli used [*C. albicans*, phorbol myristate acetate (PMA), *Staphylococcus aureus*], but no external NET structure could be observed. The ES could not degrade already-formed NETs, indicating a blockade downstream of ROS production but prior to the physical release of NETs from the cell. The mechanism of this blockade is currently unknown, but it may involve inhibition, for example, of the pore-forming protein gasdermin D, which is involved in the release of DNA across the nuclear and cellular membranes (133). The authors also show that other functions of neutrophils, such as chemotaxis and phagocytosis, are not impacted by *T. spiralis* ES products (178).

Neutrophil elastase is a key molecule that acts early on in the formation of extracellular traps and leads to DNA decondensation. Interestingly, proteomic studies in helminths, chiefly schistosomes, have identified inhibitors of neutrophil elastase (129–131). In *S. mansoni*, for example, SmKI-1, a secreted serpin, has been shown to bind neutrophil elastase and impair neutrophil migration and function in murine models of inflammatory disease. The involvement of SmKI-1 in NETosis has not been studied to date. In *T. spiralis*, another serpin was recently shown to block neutrophil elastase, and also impair phagocytosis and NETosis, and reduce the release of pro-inflammatory cytokines and chemokines (97). *Trichinella spiralis* also secretes calreticulin (*Tsp*CRT) that can bind the complement component 1q (C1q). This binding in turn was found to reduce extracellular trap formation triggered by albumin/anti–albumin complexes (in a C1q-dependent manner) *in vitro* (132).

Despite the description of eosinophil traps against helminths, to date, no specific helminth evasion mechanisms for EETs have been described, but they are likely to exist and warrant further exploration.

#### 3.2.2 Evasion from cell recruitment and effector molecules

A few mechanisms for evading direct killing by myeloid cells have also been reported.

##### 3.2.2.1 Eosinophils

The so-called Protein 1 from *Ascaris suum* (As-PAS-1), secreted at both larval and adult stages, has been shown to have anti-inflammatory properties. Notably, it abrogates both the inflammatory responses induced *in vitro* by LPS (136) and *in vivo* in the ovalbumin (OVA)-induced allergic airway mouse model (137). The mechanism underlying PAS-1 is dependent on IL-10 and T-regulatory cells (Tregs), but intriguingly, PAS-1 also directly impaired eosinophil peroxidase activity and reduced levels of IL-4, IL-5, IL-13, and eotaxin (138), demonstrating both direct and indirect mechanisms of immune evasion.

Interestingly, total schistosomal lipids or the schistosomal-derived lysophosphatidylcholine (Sm-LPC) fraction not only activate DC and macrophage polarization, as mentioned above but have also been shown to activate lipid droplet biogenesis in human eosinophils *via* TLR2 *in vitro* (140, 141). The authors further showed that this mechanism was dependent on the eicosanoid receptor DP1 (recognizing PGD2) for total schistosomal lipids. Interestingly, the mechanism was not DP1 dependent for the Sm-LPC fraction alone, indicating that it is *S. mansoni*-derived PGD2 that is responsible for this eosinophil activation phenotype (and specifically the release of three active molecules, namely EXC4, LTC4, and TGF-β).

##### 3.2.2.2 Neutrophils

Helminth ES products contain many products with redox activity, such as thioredoxins, peroxiredoxins, and superoxide dismutase (179). It has been hypothesized that these enzymes can evade or detoxify the reactive oxygen- and nitrogen-based species utilized by granulocytes. In addition, it has been shown that peroxiredoxin-1, a secreted product from *S. japonicum*, can use RNA interference (RNAi) knockdown to be protective against H_2_O_2_ exposure but not against nitric oxide (NO) exposure (180).

Two hookworm products, neutrophil inhibitory factor (NIF) from *A. caninum* and Na-ASP-2 from *N. americanus*, have been shown to have opposing impacts on neutrophil chemotaxis, with NIF inhibiting neutrophil accumulation at sites of tissue injury through CD11b/CD18 binding (134), and Na-ASP-2 recruiting neutrophils and monocytes to the skin (135).

##### 3.2.2.3 Mast cells and basophils

Histamine release is another hallmark of helminth infection, and, although it is unclear whether or not histamine is required for killing helminths, worm products can either increase or decrease histamine release from mast cells and basophils.

For example, the aforementioned ES-62 from *A. viteae* has been shown to inhibit mast cell degranulation (139) *via* its ability to “reset” the homeostatic signaling of ST2, the IL-33 receptor. Using peritoneal-derived serosal mast cells, this mechanism involves the sequestration of MyD88 to ST2, which in turn limits crosstalk with macrophages and the inflammatory cytokine cascade (181). In contrast, a translationally controlled tumor protein (TCTP) homolog from *S. mansoni* can induce histamine release from a basophil/mast cell line (182).

It is quite clear that many helminth evasion mechanisms are directed toward myeloid cells, highlighting the high risks that those cells pose to the parasites. An efficient therapy against helminths thus needs to circumvent this evasion/immunomodulation, both to restore efficient antibody production and empower myeloid cells to conduct cellular killing.

## 4 Alternatives to humoral immunity as a vaccine strategy

Epidemiological and experimental evidence does not support a strong role for humoral immunity to helminths. In this section, we discuss the correlates of immunity to helminths in the context of vaccine design.

Research in endemic settings indicates that T-cell responses to some helminths are particularly poor. In hookworm-infected patients cured with chemotherapy in China and Brazil, peripheral blood mononuclear cells (PBMCs) from individuals older than 40 years remained hyporesponsive to restimulation with hookworm antigen for up to 12 months (183). The effects of immunomodulation, therefore, worsen with age, or more likely, with chronic exposure to parasites. Furthermore, Loukas et al. (183) have observed that patients with mixed infections by schistosomes and hookworm had reduced cellular responsiveness to schistosome antigens compared to age-matched controls with single-infection schistosomiasis. Thus, hookworm in particular compromises T-cell responses directed against itself and other pathogens. Phase 2 clinical trial results for the two lead hookworm vaccine candidates have presented additional challenges to traditional vaccine design (13). The trial used mass cytometry from participants’ PBMCs in the endemic setting of Gabon to measure cellular responses to the *Na*-GST-1 antigen. Surprisingly, vaccination-induced cognate T cells expressed high levels of CTLA-4 and CD40-L, checkpoint molecules typically associated with Tregs. It is likely that these “regulatory” cells compromised immunity and led to low antibody titer results, even after three doses of the vaccine candidate. As illustrated here in the case of hookworms (but also likely for other helminths), traditional vaccines may struggle to override existing immunomodulation. Instead, antigen-specific immunity may need to be “rewired” or de-tolerized, and protection by innate cells might be preferable to a humoral response.

Lessons learned from vaccine research in agricultural and laboratory models highlight that innate cell immunity may be central to protection. Interestingly, an agricultural vaccine study against *H. contortus* demonstrates the importance of cellular responses for protection. While studying the effect of different adjuvants in an experimental L3 surface antigen vaccine, Piedrafita et al. (184) found no correlation between antibody titers and protection (as measured by fecal egg output). Instead, using an intradermal challenge model, skin immune responses and, notably, eosinophils were shown to be strongly correlated with protection (184).

Similarly, with the commercial Barbervax vaccine against *H. contortus*, antibody titers (antigen-specific and non-specific) do not necessarily correlate with vaccine-induced protection. Furthermore, this has been reported in studies of natural exposure (where sheep may have been previously exposed to the parasite) (185). A weak correlation has been observed in naive flocks (186) and even in studies where the antigen dose was 20 times higher than that of the commercial vaccine (187). Together, these findings suggest that humoral immunity alone does not mediate or predict protection against this gastrointestinal nematode.

In another agricultural vaccine against *Ostertagia ostertagi*, the number of natural killer *(*NK) cells, rather than antibody titers, was correlated with protection (188). Similarly, vaccination against *O. volvulus* ASP-1 has been shown to induce a dominant interferon-gamma (IFN-γ) response, which is likely linked to the abundance of activated NK cells and neutrophils (189). Finally, a recent vaccine against *T. spiralis* based on a recombinant galectin was shown to be protective and cause high levels of intestinal histamine release (190).

Altogether, this suggests that despite the immunomodulation of the adaptive immune system, innate cell-mediated responses can support helminth vaccinations. We propose in the following section that the trained immunity of myeloid cells could be harnessed for efficient vaccination against helminths. We first introduce the concept of trained immunity, review the current evidence of trained immunity in helminth infection, and then speculate on helminth products that could cause such myeloid cell memory. Finally, we detail the concept of a combined antigen/trained immunity vaccine against helminths.

### 4.1 Immunological basis of trained immunity

**Figure 3:**
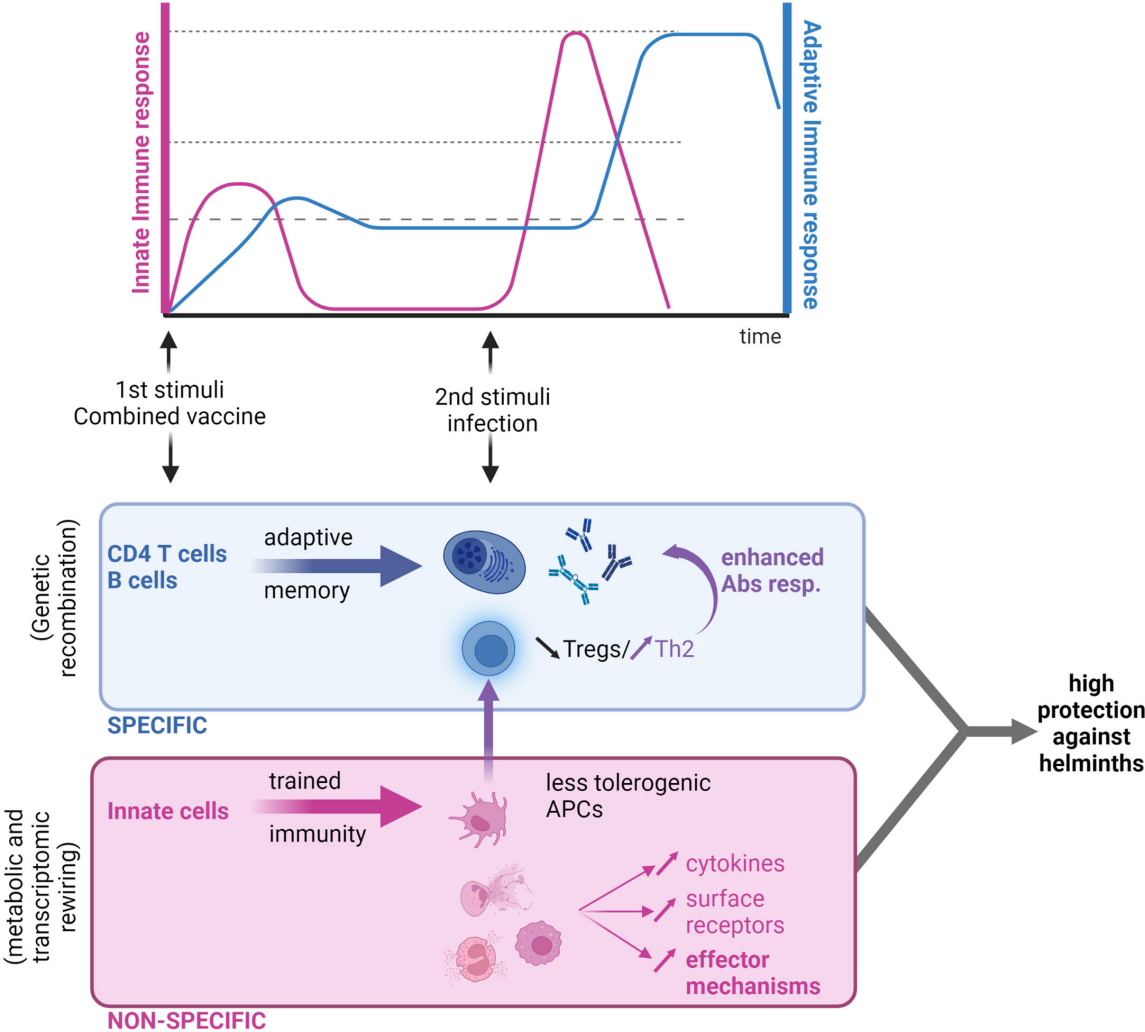
Next-generation anthelmintic vaccine: A combination of a trained immunity vaccine and a traditional antigen-based vaccine. Traditional vaccines based on parenteral antigen administration cause the development of humoral immune responses that are highly specific to the pathogen targeted (in blue). In the context of helminth infection, low T-cell activation and proliferation is a cause of concern for poor antibody responses. Trained immunity-based vaccines are based on the “education” of innate immune cells that would trigger a quick response to the same or a heterologous infection (in red). This response is however unspecific. In the context of helminth infections, the right “priming” of myeloid cells could lead to (i) an increase in effector mechanisms mediated by myeloid cells, and (ii) a “rewiring” of antigen-presenting cells (APCs) and restoration of a T-cell balance in favor of Th2 (by decreasing Treg counts), which would ultimately enhance antibody production if used in combination with a traditional vaccine approach. Figure created using BioRender.com.

The concept of innate immune memory is little more than a decade old, with the adaptive features of NK cells first reported in mice (191, 192). When “memory” NK cells were adoptively transferred from mice previously infected with cytomegalovirus into naive animals, they still conferred protective immunity typical of reinfection (193). Following this study on NK memory, Kleinnijenhuis and colleagues (194) observed epigenetic reprogramming of human monocytes after bacillus Calmette–Guérin (BCG) vaccination, which enhanced cytokine production in response to restimulation (194). In contrast to adaptive memory, which increases the specificity and quality of antigen-specific responses, “trained immunity” amplifies non-specific responses (**Figure 3**). Furthermore, circulating monocytes collected before and up to 3 months after BCG vaccination were found to be hyperresponsive not only to secondary mycobacterial stimuli but also to unrelated pathogens such as *C. albicans* and *S. aureus* (194). This sensitization is T and B cell-independent and maintained by epigenetic modifications, such as methylation of histone 3 at lysine 4 (H3K4), in regions associated with pro-inflammatory cytokine expression.

The rewiring associated with trained immunity has been shown not only on an epigenetic level but also on a metabolic level. For example, aerobic glycolysis has been identified as a basis for trained immunity in macrophages, similar to the Warburg effect observed in activated T-cells (195). Blocking H3 methylation *via* the inhibition of cyclic AMP signaling at the same time that monocytes encounter a primary stimulus (*i.e.*, the initial “training”) abrogates trained memory responses to a secondary stimulus (196). Trained immunity can also be licensed by crosstalk with adaptive cells. In the case of trained immunity against protozoan parasites, adherent peripheral blood mononuclear cells (*i.e.*, monocytes and DCs) stimulated with *Plasmodium falciparum*-infected red blood cells (iRBCs) are trained to hyperproduce cytokines following a secondary TLR2 stimulus (197). The authors have shown *in vitro* that this training is dependent on the presence of T-cells during the initial exposure, which is most likely to provide IFN-γ and induce programmed death ligand 1 (PD-L1) expression in monocytes and DCs. Importantly, similar epigenetic modifications have been found in the circulating monocytes and DCs of malaria patients.

Myeloid cells are typically not long-lived, and due to this, there have been doubts as to the usefulness of this short-term trained immunity for vaccination purposes. However, it has recently been shown that trained immunity can affect some longer-lived cells, such as fibroblasts (198) and hematopoietic stem cells (HSCs) (199). HSC training has been proposed to be a form of “centrally trained immunity”.

Niche-specific mucosal training has also recently been demonstrated and may be of particular importance for an anthelmintic vaccine in these tissues. For example, using fluorescent tracking, it has been shown that alveolar macrophages are primed by BCG exposure independently of circulating monocytes and HSCs. Interestingly, the authors also showed that this priming was only weakly inhibited in the presence of methylation inhibitors, indicating that a circulating metabolite underpins the observed priming (200). They further characterized the metabolites as the microbiome-derived short-chain fatty acids deoxycarnitine and butyrate and proved that supplementation of those metabolites in drinking water can mimic macrophage training to protect mice from *Mycobacterium tuberculosis* infection, raising the very interesting possibility of metabolic supplementation to induce trained immunity (200).

The field of trained immunity is still young, but it is already quite apparent that mucosal, distal, or centrally trained immunity can all be harnessed to fight helminth infection.

### 4.2 Helminth evidence of trained immunity: Myeloid cells and beyond

In recent years, a few studies have described the trained immunity induced by both nematode and trematode infections. Given that this is an emerging field with a limited number of studies, we report all of them here rather than those only pertaining to myeloid cells and argue that if trained immunity is present in some innate cells, it may be present in others.

Two studies have focused on the innate training of macrophages. Quinn et al. (201) use *F. hepatica* ES products to train bone marrow-derived macrophages *in vitro* (201). After a rest period, macrophages were restimulated with various pro-inflammatory triggers, such as LPS and Pam3CSK. Interestingly, the inflammatory response of Fh-ES-trained macrophages was lower than that of naive macrophages. In fact, after LPS stimulation, IL-10 release was increased. The authors then showed that treatment with a methyltransferase was able to reverse this Fh-ES-induced training, demonstrating an epigenetic basis for this macrophage profile (201). Similarly, the soluble fraction of the crude extract of *T. suis* was shown to suppress pro-inflammatory cytokines released by bone marrow-derived macrophages stimulated with LPS (202). Training macrophages with *T. suis* extract compromised inflammatory responses to a wide range of triggers (*e.g.*, CpG, LPS, and Pam3CSK) but, interestingly, enhanced IL-4 polarization, as measured by increases in CD200 and CD206. The authors further showed that this training had a metabolic basis, with a shift toward higher levels of oxidative phosphorylation.

Those studies show trained immunity in the context of tolerance induction, but others have also shown a more traditional trained immunity response which induces protection against the parasite. Pionnier et al. described Nkp46^+^ NK cells as the major innate lymphoid population recruited at the site of infection with *B. malayi* (203). In *Rag*2 knockout mice, the depletion of NKp46^+^ cells increased susceptibility to infection. The authors further showed that the long-term activation of NKp46^+^ cells plays a protective role during secondary infection in *Rag*2 knockout mice, proving that memory is intrinsic to the innate compartment (203).

Interestingly, and probably of utmost importance for vaccine design, innate training by helminths is not limited to short-term impacts. Indeed, it was recently shown that treating mice with Fh-ES also imprints a long-lasting memory on hematopoietic stem cells (HSCs) in the bone marrow through metabolic and transcriptional rewiring (204). Mice treated with Fh-ES had enhanced proliferation and expansion of myeloid-committed precursors, resulting in the expansion of anti-inflammatory monocytes. This helminth-induced anti-inflammatory trained immunity rendered the mice less susceptible to the induction of experimental autoimmune encephalomyelitis, a mouse model of multiple sclerosis. Another long-term effect was seen in maternal schistosome exposure during the perinatal period, which has been shown to confer protection against asthma in the next generation, but also a reduced capability of infants to respond to vaccination. Lacorcia et al. investigated the mechanisms behind such epidemiological observations in mice and associated them with a persistent change in the activation profile of antigen-presenting cells (205). Furthermore, they showed higher CD86 expression in both conventional DC subsets 1 and 2, and also in F4/80^+^ cells, whereas isolated splenic DCs from maternally exposed mice were less able to cause T-cell proliferation.

The gut–lung axis is crucial to immunity during intestinal helminth infection. In line with the recent finding of distal training of alveolar macrophages, it was recently reported that prior exposure to *S. venezuelensis* can confer heterologous protection against *N. brasiliensis* 3 months later (36). Using ILC2-deficient Rora^sg/sg^ bone marrow chimera mice, the authors further demonstrated that pulmonary ILC2s were required for this protective mechanism. CD4+ T-cell depletion before *N. brasiliensis* infection did not affect protection, nor did it alter the number of ILC2s present in the lungs. Overall, this paper proves the reprogramming of ILC2s by helminths and their potential for cross-protection against other helminth species. Of note is that the effector cells seem to be eosinophils and not ILC2s, as IL-5 blockade completely abrogated protection.

All in all, recent studies clearly illustrate that, as in allergic disease (206), helminth infection induces reprogramming of the innate compartment that participates in the fine-tuning of the type 2 immune response, with both protection and tolerance induction. In the context of vaccine design, this means trained type 2 immune responses could be harnessed, or at the very least, trained regulatory immunity must be understood and overcome.

### 4.3 Other potential trained immunity inducers in helminth infection

Even if not investigated to date, several key components of helminth biology could trigger innate, trained immunity and should be studied further.

#### 4.3.1 Pathogen-associated molecular patterns and damage-associated molecular patterns

Pathogen-associated molecular patterns and damage-associated molecular patterns (PAMPs and DAMPs, respectively) are inducers of trained immunity [as reviewed in Jentho and Weis (207)].

The most well-characterized PAMPs associated with trained immunity are β-glucans, a heterogeneous component of many pathogens such as yeast, bacteria, and fungi that are not produced by mammalian cells (208–211).

There are no clear PAMPs associated with helminth recognition; instead, DAMPs have been shown to play a primordial role in the initiation of anti-helminth type 2 responses. However, helminth parasites are rich in glycans, and many glycoproteins are involved in immunomodulation [ (212); Section 3]. Whether or not β-glucans are specifically, which have been shown to induce trained immunity, produced by helminths is not formally proven. However, we have recently demonstrated that laminarin supplementation competitively inhibits the binding of macrophages to *N. brasiliensis* larvae in a pathway dependent on CD11b or the β-glucan receptor Eph2A (213). Another β-glucan receptor, dectin-1, has also been shown to be important for the recognition of egg antigens from *S. mansoni* and triggers the release of ETs in response to microfilariae. Dectin-1 is also a target for immunomodulation mediated by *Hpb* and *F. hepatica* (83, 91, 113, 214, 215). Altogether, this suggests that β-glucans on helminths, or in close association with them (*i.e.*, fungal or bacterial β-glucans from the helminth microbiome), could play a role in training the innate immune system. Of note is that other PAMPs of bacterial or fungal origin from microbiome-associated or endosymbiotic bacteria/fungi could also potentially be part of the helminth-induced trained immunity.

Heme, along with the detoxification molecule hemozoin released by *Plasmodium*, is one of the DAMPs that have been shown to trigger trained immunity (216). Owing to their large size, helminths can cause hemorrhages, and several helminth blood feeders detoxify heme through hemozoin (*S. mansoni* and *N. brasiliensis*). As such, heme and its derivatives could be a potential trigger for innate immune training in the context of helminth infection. In the context of allergy, it has recently been shown that IL-33 was participating in the training of ILC2s (217, 218). As IL-33 is central to helminth infection control (219), it would be extremely interesting to determine if IL-33 is participating in the training of ILC2s and macrophages for the control of helminth infection.

#### 4.3.2 EVs in trained immunity

Because they convey various molecules and are taken up by host cells in the immediate vicinity, parasite EVs appear as logical players in trained immunity. However, evidence that EVs can indeed induce trained immunity is limited, although it has recently been shown that EVs from intestinal bacteria can cause trained immunity in bone marrow-derived macrophages *in vitro*, and also decrease the IL-10 and TNF responses to LPS Stimulation (220). EVs from monocytes containing the HIV component Nef (negative factor) were also shown to cause long-term hyperactivity in monocytes in an epigenetic and metabolic rewiring-dependent mechanism (221).

Although trained immunity has not directly been investigated in this study, Coakley et al. observed that the vaccination of mice with *Hpb* EVs and alum decreased worm burdens. Most strikingly, this protection was not antibody-mediated, as ST2 knockout mice were not protected by vaccination, despite having elevated IgM, IgA, and IgG1 titer results (170). The authors further show that *Hpb* EVs could block the differentiation of bone marrow-derived macrophages into AAMs, with EV–macrophage cocultures having lower gene expression levels of *Ym1*, *RELMα*, and *arginase-1*, and *CCL17*. Anti-EV antibodies increased the uptake of EVs into cells but diverted them to the lysosome and protected cells from immunosuppression, suggesting that neutralizing antibodies against EVs are capable of blocking immune evasion, allowing natural innate immunity to clear a primary helminth infection. However, in different helminth infection models, EV-based vaccination has differing degrees of success at reducing worm burden, as reviewed by Drurey et al. (163).

EVs have been demonstrated to be taken up by many different myeloid cells. For instance, ES miRNAs from *L. sigmodontis* were preferentially detected in macrophages *in vivo* (162), similar to EV-encased miRNAs from *S. japonicum* (161). The latter was shown to have the ability to regulate host macrophage functions *via* incorporation into mouse argonaute 2 (Ago2, a component of the RNA-induced silencing complex) *in vitro*, a prerequisite for them to exert their silencing function. Overall, EVs from *S. japonicum* induce an M1-type immune profile in macrophages *in vitro* (222), as was observed with *B. malayi* EVs (102).

Eosinophils, too, accumulate at the site of infection and are exposed to large amounts of helminth EVs. *In vitro*, human eosinophils exposed to *S. mansoni* adult lipid extracts were directly activated, eliciting the syntheses of leukotriene C4 and eoxin C4 and also the secretion of preformed TGF-β. The main eosinophil-activating components within *S. mansoni* lipids were identified as schistosomal lysophosphatidylcholine and PGD2, directly acting on eosinophil TLR2 and DP1 (141). Similarly, macrophages exposed to schistosomal lysophosphatidylcholine polarized toward the AAM phenotype and produced IL-10, TGF-β, and PGE2 through a PPARγ-dependent mechanism (121). Similarly, schistosomal lysophosphatidylserine stimulated DC maturation *via* TLR2 and induced IL-10-producing Tregs (223). Overall, *S. mansoni* EV lipid components were proposed to not only confer structural packaging properties to EVs but also mediate immunomodulation directly (122, 224).

Altogether, this suggests that helminth EVs could be involved in the long-term immunomodulation of myeloid cells, potentially through innate training mechanisms.

### 4.4 Trained immunity in helminth infection: a path to long-term control?

The current vaccine design against helminths focuses on the adaptive immune system; however, helminths induce low levels of T-cell proliferation and an overall dampening of immunity. Recent studies have shown that anthelmintic treatment can abrogate tolerance mechanisms; however, in a similar process to trained immunity, helminths can induce long-term “tolerance” and immunomodulation that persists after parasite clearance. We thus propose that the new generation of vaccines against helminths should integrate innate and adaptive immune memory (**Figure 3**).

The antigen-based part of the vaccine would confer long-lasting and specific memory, whereas the trained immunity part of the vaccine would focus on (i) abrogating tolerance by re-educating antigen-presenting cells, thus allowing for efficient T-cell expansion, and (ii) boosting non-specific myeloid-, innate lymphoid-, and non-immune-based memory in a non-specific but “tailored” manner.

Combined vaccines (*e.g.*, those with multiple antigens) present a considerable regulatory challenge, which often hampers the development of medicines that will primarily be used in low- and middle-income countries. This is in part because costly safety and dose-finding studies must assess each antigen separately and in combination. However, as trained immunity has recently been shown to be mediated by metabolites and other non-antigenic compounds, we suggest an approach in which “trained immunity” adjuvants, such as β-glucans, are combined with existing helminth antigens.

Of note is that complications and problems linked to the design of trained immunity-based vaccines are not well defined as yet due to the relatively recent emergence of this field. One limitation of the trained immunity-based vaccines currently used for humans is their partial effectiveness. For example, approximately only half of individuals who receive the BCG vaccine respond with strong trained immunity (225, 226). In addition, most collected data are epidemiological in nature rather than coming from a controlled clinical trial, and, as such, could suffer from intrinsic bias. Another potential limitation is the impact of bystander antigens or infections. Indeed, BCG or measle vaccination, are now well accepted to confer nonspecific protection against infantile infections, especially in low income and middle-income countries (227). However, whether an anti-helminth-trained immunity would also be advantageous or would at least not interfere with current protection against childhood diseases will need to be investigated, and advancements in this field should be made cautiously.

Further exploration of the immune training caused by helminths may thus offer new opportunities for the control of these parasites. In particular, it may be possible [as it is currently proposed for allergy (206) and other non-communicable diseases (228)] to overcome the epigenetic and metabolic rewiring that helminth immunomodulation imposes on the host.

## 5 Conclusions

Considerable global achievements have been made in controlling helminth infections using chemotherapeutic interventions and poverty-reduction measures such as improved water, sanitation, and hygiene services. However, mass drug administration programs do not protect against reinfection, and thus are not a long-term solution to helminth infection and require continuous public health efforts, even in low-transmission settings to maintain adequate levels of coverage and compliance (229, 230). Developing an HHV has therefore been an important global research goal of the last two decades. Here, we have reviewed a large number of recent findings that implicate non-humoral and innate immunity as key components in the control of helminth infection, both in experimental models and in agricultural trials. While current HHVs aim to reduce morbidity (and not necessarily reduce worm burden), we believe that better harnessing of myeloid cells in future vaccines could improve their efficacy and in turn help to break the cycle of transmission. Trained immunity offers an exciting approach to achieving this for several reasons. First, it has a demonstrated ability to activate immune cells at mucosal sites, such as the lung, where sterilizing immunity to some soil-transmitted helminths occurs in animal models. Second, despite its non-specific nature, different metabolic and epigenetic mechanisms underpin different types of trained immunity, potentially allowing a trained immunity vaccine to be targeted in its design. Third, while not always referenced in the literature, trained immunity has in fact been demonstrated to occur during helminth infection, and several models of vaccination with ES products and EVs protect mice from infection. Given that trained immunity can be induced by metabolites and other products, the use of these unrelated biomolecules would be more technically feasible than vaccines that require scarce worm-derived products (as is the case with Barbervax), which in some cases makes it impossible to meet Good Manufacturing Practice standards. All in all, we have made a case for trained immunity to be considered in ongoing research efforts for HHVs.

## Author contributions

RD, TB, and LT conceived the idea of the review and wrote the document. NP worked on the literature search and prepared the figures for the review. All authors contributed to the article and approved the submitted version.
